# Sachet water consumption as a risk factor for cholera in urban settings: Findings from a case control study in Kinshasa, Democratic Republic of the Congo during the 2017–2018 outbreak

**DOI:** 10.1371/journal.pntd.0009477

**Published:** 2021-07-08

**Authors:** Placide Mbala-Kingebeni, Florian Vogt, Berthe Miwanda, Tresor Sundika, Nancy Mbula, Isaac Pankwa, Leopold Lubula, Veerle Vanlerberghe, Alain Magazani, Mildred Tita Afoumbom, Jean-Jacques Muyembe-Tamfum

**Affiliations:** 1 Institut National de la Recherche Biomédicale, Kinshasa, Democratic Republic of the Congo; 2 Institute of Tropical Medicine Antwerp, Antwerp, Belgium; 3 National Centre for Epidemiology and Population Health, Research School of Population Health, College of Health and Medicine, Australian National University, Canberra, Australia; 4 The Kirby Institute, University of New South Wales, Sydney, Australia; 5 FELTP DRC, Kinshasa, Democratic Republic of the Congo; 6 DGLM DRC, Kinshasa, Democratic Republic of the Congo; 7 Hasselt University, Hasselt, Belgium; Wayne State University, UNITED STATES

## Abstract

**Background:**

Behavioural risk factors for cholera are well established in rural and semi-urban contexts, but not in densely populated mega-cities in Sub-Saharan Africa. In November 2017, a cholera epidemic occurred in Kinshasa, the Democratic Republic of the Congo, where no outbreak had been recorded for nearly a decade. During this outbreak, we investigated context-specific risk factors for cholera in an urban setting among a population that is not frequently exposed to cholera.

**Methodology/Principal findings:**

We recruited 390 participants from three affected health zones of Kinshasa into a 1:1 matched case control study. Cases were identified from cholera treatment centre admission records, while controls were recruited from the vicinity of the cases’ place of residence. We used standardized case report forms for the collection of socio-demographic and behavioural risk factors. We used augmented backward elimination in a conditional logistic regression model to identify risk factors.

The consumption of sachet water was strongly associated with the risk of being a cholera case (p-value 0.019), which increased with increasing frequency of consumption from rarely (OR 2.2, 95% CI 0.9–5.2) to often (OR 4.0, 95% CI 1.6–9.9) to very often (OR 4.1, 95% CI 1.0–16.7). Overall, more than 80% of all participants reported consumption of this type of drinking water. The risk factors funeral attendance and contact with someone suffering from diarrhoea showed a p-value of 0.09 and 0.08, respectively. No socio-demographic characteristics were associated with the risk of cholera.

**Conclusions/Significance:**

Drinking water consumption from sachets, which are sold informally on the streets in most Sub-Saharan African cities, are an overlooked route of infection in urban cholera outbreaks. Outbreak response measures need to acknowledge context-specific risk factors to remain a valuable tool in the efforts to achieve national and regional targets to reduce the burden of cholera in Sub-Saharan Africa.

## Author summary

Cholera is a diarrheal disease caused by ingestion of the *Vibrio cholerae* bacterium. Outbreaks in urban areas are becoming increasingly frequent in Sub-Saharan Africa. Risk factors for cholera have been studied in rural settings but not sufficiently in urban areas. Understanding context-specific risk factors is key for successful outbreak response. During a cholera outbreak in Kinshasa, the Democratic Republic of the Congo we were able to identify a previously unknown behavioural risk factor of particular relevance in urban settings–the consumption of drinking water from plastic sachets. Water sachets are sold on the streets of all major cities in Sub-Saharan Africa. It requires biting off an edge and sucking out the water, and we think that external contamination of these sachets was an important transmission route in the Kinshasa outbreak. Water sachets are predominantly consumed by socio-economically disadvantaged groups who lack piped water supply in their homes and have poor access to sanitary infrastructure. This makes our findings particularly relevant because these are the very populations who are at increased risk of getting and transmitting cholera. Health messaging and response measures should include consumption of water sachets as a potential risk factor during future cholera outbreaks in urban low-resource settings.

## Introduction

Cholera is an infectious disease caused by the *Vibrio cholerae* bacterium through the ingestion of faeces-contaminated water or food. Transmission is either direct through exposure to an infectious individual or indirect through faecal contamination in the broader environment. Humans are the only host but *V*. *cholerae* can also survive in open water [1]. Typical symptoms are sudden onset diarrhoea, stomach cramps and vomiting, leading to quick dehydration and potentially death if left untreated. About 20% of infected people become symptomatic, of which the majority remain mild and only 20% continue to develop severe symptoms. Case fatality among symptomatic patients can reach 50% in the absence of treatment but can be brought down to below 1% through early rehydration and, to a lesser extent, antibiotic treatment [2,3].

There are an estimated 1.3–4.0 million cholera cases each year globally, both in endemic areas as well as through outbreaks after introduction in non-endemic areas [4]. Cholera has been endemic in the East of the Democratic Republic of the Congo (DRC) at least since the mid-1970s and accounts for 5%-15% of all cholera cases worldwide [4]. Specific enabling factors such as civil war, breakdown of public infrastructure, high population movement and high population density aggravate other common deficiencies of the public health sector in DRC that enhance cholera outbreaks such as lack of waste water management and access to safe drinking water [5].

As in many Sub-Sahara African (SSA) countries, most cholera cases in DRC occur in rural or semi-rural areas confined to so-called “hotspots”, i.e. small geographical areas with high concentrated cholera incidence [6,7]. These hotspots are located mostly in the eastern part of DRC, where several major lakes function as environmental reservoirs. However, an epidemic that originated from Kindu (Maniema province, Eastern DRC) in mid-2015 slowly started progressing westwards along the major rivers during the course of 2016 and 2017, eventually spreading into 24 out of all DRC’s 26 provinces, many of which had been cholera-free for over 10 years [8]. This epidemic caused 55,000 cases and 1,190 deaths along its way, before reaching the capital of Kinshasa in November 2017. Over the next 3 months, more than 1,000 cases were recorded in Kinshasa with a case fatality ratio of 4% [9].

A number of general “classic” risk factors for cholera that are related to direct contact with diseased cases or faeces-contaminated food and water are well established, such as using open sources for drinking water; lack of latrine use, hand washing and personal hygiene measures; caring for symptomatic patients; and poor food protection and processing [10,11]. Most of this existing evidence originates from rural, semi-rural or peri-urban populations, and in particular from South Asia, while recent studies from outbreaks in SSA mega-cities such as Kinshasa remain scarce. However, cholera transmission dynamics are known to be different in urban SSA settings [12,13], and further urbanisation in combination with climate change will make urban cholera outbreaks more likely in these settings in the future [14,15]. At the same time, the World Health Organisation (WHO) has embarked on a roadmap to eliminate cholera in 20 countries by 2030 [7]. In DRC, the elimination of cholera was officially declared the aim of national health policy in 2007 and several strategic documents have been produced since then [16–18]. However, the resurgence and prolonged persistence of cholera in previously non-endemic areas of DRC shows that strategic planning alone does not eliminate cholera without, among other factors, having a good contextual understanding of transmission patterns to guide targeted outbreak control measures.

Therefore, it is paramount to have a better understanding of context-specific risk factors for cholera in Kinshasa and similar settings in SSA. We seized the 2017–2018 cholera outbreak in Kinshasa as an opportunity to investigate context-specific risk factors in a congested urban SSA setting among a population that is not frequently exposed to cholera to inform response to future outbreaks in similar contexts.

## Methods

### Ethics statement

We obtained approval from the Ethics Review Board of Kinshasa University, Kinshasa, DRC (approval number EST/CE/231) and from the Institutional Ethics Review Board of the Institute of Tropical Medicine Antwerp, Belgium (reference number 1316/19). Individual written informed consent was sought from all participants and from parents or guardians for participants below 18 years of age.

### Design

This was a case-control study with 1:1 matching by age and proximity of place of residence, aiming to identify risk factors for cholera among facility-based cases and community-based controls.

### Setting

Kinshasa, located in the Western part of DRC, is home to about 11 million inhabitants and features several characteristics that are both typical for many SSA megacities as well as conducive to spark and aggravate cholera outbreaks. Rapid population growth and outmigration from rural areas led to large informal settlements of substandard housing with high population density, often located in flood-prone areas, that lack sewage and waste water management, garbage disposal and clean water supply. This study was conducted in the three health zones that were hit hardest during the outbreak, namely Binza Météo, Limete and Kintambo. Limete is located in central Kinshasa close to the Congo river with substantial cargo and passenger traffic to and from other parts of the country by boat, while Kintambo is located in the north-west and Binza Météo in the central-eastern area of Kinshasa. All three areas are characterised by crowded living conditions, high population mobility, lack of safe drinking and sewage water infrastructure, and poor housing quality.

### Study population

For the purpose of this study we defined cases as all persons admitted to the cholera treatment centres (CTCs) in Limete between 1 and 28 February 2018 with place of residence in the health zones of Limete, Kintambo or Binza Météo. Admission criteria at the CTC Limete was presentation of acute watery diarrhoea with or without vomiting, thereby following national guidelines for the cholera case definition during outbreaks [19]. As common practice during cholera outbreaks, culture-based laboratory confirmation was only done at the beginning of the outbreak. Out of 177 stool samples collected between November 2017 and March 2018, 83 tested positive for *V*. *cholerae* by rapid diagnostic test [9].

We identified controls among inhabitants of the compound that was closest to the case’s compound and in which no cholera case had occurred during the outbreak, who did not report any diarrhoeal symptoms since the beginning of the outbreak in Kinshasa. Controls had to fall within a 5-year age range of the respective case. If no eligible person was identified in the nearest compound, we moved to the second-closest compound of the case. If in a compound there was more than one eligible control within the case’s 5-year age range, the person that matched the case’s age most closely was selected as control.

### Data collection

Data collection was conducted between 8–30 February 2018 using as data sources CTC patient registers for case identification and household survey questionnaires administered to cases and controls for risk factor identification. See S1 Table for variable definition and measurement. Field work was carried out by trained staff from the national field epidemiology training programme (AFENET), following pre-defined standard operating procedures (SOP) and using pre-tested case report forms (CRF). Senior epidemiologists from AFENET and INRB provided on-site supervision.

### Sample size

Assuming 25% exposure among controls and aiming to detect a minimum odds ratio of 2 with 90% power and an alpha risk of 5% required a total sample size of 402 (201 cases and 201 controls).

### Data analysis

We used the software package SAS v9.4 for our data analysis. We produced two- and three-way contingency tables and calculated Cochran-Mantel-Haenszel odds ratios (OR) for data exploration purposes and for descriptive analysis. We then built a conditional logistic regression model to identify risk factors for cholera. For this, we opted for the Augmented Backward Elimination (ABE) method, which combines significance levels as well as changes in parameter estimates based on the Akaike Information Criterion (AIC) for variable selection [20]. The full model was fitted by removing the least significant variable based on 5% significance levels iteratively while screening for changes in the AIC. A variable that caused an AIC change of at least 15% was retained in the model, and the next least significant variable was checked subsequently. This process continued until no more variables could be removed from the model. We used Generalized Variance Inflation Factor (GVIF) analysis to investigate multicollinearity between selected variables [21].

We used ORs and 95% confidence intervals (95% CI) to assess the strength of associations and precision of point estimates, and p-values for hypothesis testing. Following widespread practice, we used alpha errors of 0.05 to classify variables as “statistically significant”. We calculated proportions of missing data for variable and used multiple imputation based on Fully Conditional Specification (FCS) [22]. To assess the robustness of the Missing At Random assumption, we performed sensitivity analyses by fitting a Pattern Mixture Model based on the Complete Case Missing Value method as well as the Subgroup Adjustment method [23].

## Results

After data cleaning and verification, we were able to use 390 observations from 195 pairs of 1:1 matched cases and controls for analysis, and constructed 20 variables from the information collected from CRFs. While the sex ratio was balanced between cases and controls, 225 (57.7%) of participants were female. While socio-economic characteristics did not differ substantially between cases and controls, univariate analysis suggested cases were more likely to have attended a funeral recently (7.5% vs 4.8%), less likely to not have consumed food at the roadside (19.8% vs 25.3%), and less likely to have washed fruit before consumption (40.3% vs 48.0%). Notably, more than 80% of all participants reported consumption of street vended sachet water. Controls were more likely to not have consumed this type of drinking water (23.5% vs 16.7%), while cases reported higher “often” or “very often” consumption than controls (52.1% vs 44.4%) (Table 1). Age, not a risk factor for analysis due to the matched study design, was evenly distributed among study participants with a mean of 23.1 years (SD 17.0), with 20–30 years olds accounting for 91 (23.0%) of all participants (S1 Table).

**Table 1:** Univariate analysis.

	Cases (N,%)	Controls (N,%)	CMH OR (95% CI)
**Sex**	**N = 195**	**N = 195**	
Male	82 (42.1)	83 (42.6)	n.a.
Female	113 (58.0)	112 (57.4)	1.0 (0.6–1.7)
**Religion**	**N = 190**	**N = 185**	
None	8 (4.2)	15 (8.1)	n.a.
Catholic	25 (13.2)	21 (11.4)	-
Protestant	24 (12.7)	18 (9.7)	-
Revival	132 (69.8)	131 (70.8)	1.7 (0.5–5.6)
**Level of education**	**N = 195**	**N = 192**	
Secondary	72 (36.9)	57(29.7)	n.a.
Primary	62 (31.8)	70 (36.5)	0.7 (0.3–1.5)
None	61(31.3)	65 (33.7)	0.1 (0.0–0.8)
**Occupation**	**N = 195**	**N = 195**	
Working	41 (21.0)	37 (19.0)	n.a.
Jobless	105 (53.9)	110 (56.4)	1.5 (0.7–3.2)
pupil/student	49 (25.1)	48 (24.6)	0.2 (0.0–0.8)
**Household size**	**N = 195**	**N = 195**	
less than 3	81 (41.5)	86 (44.1)	n.a.
3 or more	114 (58.5)	109 (55.9)	0.9 (0.5–1.4)
**Presence of soap in the household**	**N = 195**	**N = 193**	
No	31 (15.9)	25 (13.0)	1.1 (0.4–3.3)
yes and confirmed^1^	127 (65.1)	118 (61.1)	
yes but not confirmed^1^	37 (19.0)	50 (25.9)	0.7 (0.3–1.3)
**Presence of toilet in the household**	**N = 193**	**N = 191**	
toilet without faecal waste present, confirmed^1^	95 (49.0)	95 (49.7)	n.a.
no toilet	80 (41.8)	81 (42.4)	0.8 (0.2–3.3)
toilet with faecal waste present, confirmed^1^	18 (9.3)	15 (7.9)	2.5 (0.4–26.2)
**Travelled outside Kinshasa since start of epidemic**	**N = 195**	**N = 191**	
No	192 (98.5)	187 (97.9)	n.a.
Yes	3 (1.5)	4 (2.1)	0.7 (0.0–5.8)
**Attended funeral since start of epidemic**	**N = 188**	**N = 187**	
No	174 (92.6)	178 (95.2)	
Yes	14 (7.5)	9 (4.8)	1.4 (0.5–3.9)
**Contact with diarrheal patient since start of epidemic**	**N = 194**	**N = 192**	
No	125 (64.4)	109 (56.8)	n.a.
Yes	69 (35.6)	83 (43.2)	0.7 (0.5–1.1)
**Health zone of area of residence**	**N = 195**	**N = 195**	
Limete	55 (28.2)	55 (28.2)	n.a.
Binza Météo	102 (52.3)	103 (52.8)	-
Kintambo	38 (19.5)	37 (19.0)	-
**Soil condition in area of residence**	**N = 193**	**N = 189**	
Dry	26 (13.5)	26 (13.8)	n.a.
Wet, humid, marshy	22 (11.4)	21 (11.1)	-
along river	145 (75.1)	142 (75.1)	1.0 (0.0–13.8)
**Source of drinking water of the household**	**N = 195**	**N = 193**	
Protected	165 (84.6)	166 (86.0)	n.a.
Unprotected	30 (15.4)	27 (14.0)	2.0 (0.4–12.4)
**Drinking water storage**	**N = 194**	**N = 190**	
closed container	188 (96.9)	185 (97.4)	n.a.
open container	6 (3.1)	5 (2.6)	2.0 (0.1–13.0)
**Sachet water consumption**	**N = 192**	**N = 187**	
No	32 (16.7)	44 (23.5)	n.a.
yes, rarely	60 (31.3)	60 (32.1)	3.3 (1.0–13.7)
yes, often	84 (43.8)	68 (36.4)	1.8 (0.5–6.8)
yes, very often	16 (8.3)	15 (8.0)	-
**Place of fruit/food purchase**	**N = 171**	**N = 168**	
market, supermarket	62 (36.3)	59 (35.1)	n.a.
roadside, street, restaurant	109 (63.7)	109 (64.9)	1.4 (0.7–3.1)
**Roadside food consumption**	**N = 192**	**N = 190**	
No	38 (19.8)	48 (25.3)	n.a.
yes, rarely	27 (14.1)	34 (17.9)	1.4 (0.4–5.6)
yes, often	118 (61.5)	103 (54.2)	2.6 (0.9–9.3)
yes, very often	9 (4.7)	5 (2.6)	-
**Procedure before food consumption**	**N = 156**	**N = 146**	
Heat	113 (72.4)	100 (68.5)	n.a.
None	43 (27.6)	46 (31.5)	0.9 (0.4–1.9)
**Roadside fruit consumption**	**N = 131**	**N = 122**	
No	77 (39.7)	66 (35.3)	n.a.
yes, rarely	3 (1.6)	2 (1.1)	0.8 (0.4–1.4)
yes, often	35 (18.0)	39 (20.9)	1.7 (0.3–10.7)
yes, very often	16 (8.3)	15 (8.0)	-
**Procedure before fruit consumption**	**N = 119**	**N = 123**	
wash with water	48 (40.3)	59 (48.0)	n.a.
wipe with hands	10 (8.4)	7 (5.7)	3 (0.2–157.5)
None	61 (51.3)	57 (46.3)	5 (0.5–24.5)

Distribution of risk factors among participants and univariate associations with risk of cholera.

^1^ Confirmed by visual inspection through the field data collector in the household.

Abbreviations: N Number; % Percentage; CMH OR Cochrane Mantel Haenszel Odds Ratio; CI Confidence Interval; n.a. not applicable.

After applying ABE, 9 of the 20 variables were retained in the final conditional logistic regression model, of which only sachet drinking water consumption showed a strong association with the risk of being a cholera case (p-value 0.019). Among those who consumed street vended sachet water, the risk of cholera increased with increasing frequency of consumption from rarely [OR 2.2, 95% CI 0.9–5.2] to often [OR 4.0, 95% CI 1.6–9.9] to very often [OR = 4.1, CI 1.0–16.7]. Having attended a funeral and having had recent contact with a diarrhoea patient had a p-value of 0.09 and 0.08, respectively (Table 2).

**Table 2:** Multivariate analysis.

	OR (95% CI)	P-value^a^	P- value^b^	Percentage missing (%)
**Religion**			0.163	4.1
None	1	n.a.		
Catholic	4.8 (1.1–20.4)	0.033^c^		
Protestant	4.0 (0.9–18.1)	0.069		
Revival	3.3 (0.9–11.1)	0.051		
**Attended funeral since start of epidemic**			0.091	3.8
No	1	n.a.		
Yes	2.6 (0.9–8.1)	0.107		
**Contact with diarrheal patient since start of epidemic**			0.083	1.0
No	1	n.a.		
Yes	0.6 (0.4–1.0)	0.078		
**Level of education**			0.118	0.77
Secondary	1	n.a.		
Primary	0.5 (0.3–1.0)	0.062		
None	0.5 (0.1–1.1)	0.085		
**Source of drinking water of the household**			0.284	0.5
Protected	1	n.a.		
Unprotected	2.2 (0.5–10.1)	0.311		
**Sachet water consumption**			0.019^c^	2.8
No	1	n.a.		
yes, rarely	2.2 (0.9–5.2)	0.067		
yes, often	4.0 (1.6–9.9)	0.002^c^		
yes, very often	4.1 (1.0–16.7)	0.046^c^		
**Place of fruit/food purchase**			0.594	13.1
market, supermarket	1	n.a.		
roadside, street, restaurant	0.9 (0.4–1.9)	0.859		
**Procedure before food consumption**			0.138	22.6
Heat	1	n.a.		
None	0.6 (0.3–1.3)	0.196		
**Procedure before fruit consumption**			0.465	37.9
wash with water	1	n.a.		
wipe with hands	1.4 (0.5–4.2)	0.558		
None	1.4 (0.6–3.7)	0.449		

Risk factors retained in the final regression model using augmented backwards elimination and adjusted associations with risk of cholera.

^a^ stratum-specific

^b^ overall.

^c^ below the pre-determined statistical significance threshold of 0.05.

Abbreviations: OR Odds Ratio; CI Confidence Interval.

While 52.0% of participants had complete information on all variables, only three variables had more than 5% missing data. We obtained the same results for both methods of sensitivity analyses (Complete Case Missing Value and Subgroup Adjustment) as in multiple imputation (S3 and S4 Tables). No issues with multicollinearity between selected variables were detected (S5 Table).

## Discussion

This was the first risk factor analysis for cholera in Kinshasa and one of few existing prospective studies from an urban Sub-Saharan African setting. We identified the consumption of street-vended sachet water as an important risk factor during the 2017–2018 Kinshasa outbreak. The detected dose-response effect, with increasing frequency of sachet water consumption being positively associated with increasing risk of cholera, together with the very high proportion of sachet water consumption overall makes us confident that this observed effect is real.

Our main finding might appear counter-intuitive at first sight since industrial processing of disinfected water into vacuum-sealed plastic sachets should eliminate most contamination risks, while untreated and unprotected open drinking water source is a well-established classical risk factor for cholera in numerous studies [10,11]. However, there is sporadic evidence that backs up our findings: Though *V*. *cholerae* has once been isolated from water sachets, albeit in low quantities, during a cholera outbreak in Nigeria in 2011 [24], it seems that sachet water quality at source or during processing and packaging is not the main concern. Several studies showed that, while bacterial contamination is not uncommon, the quality of the water inside the sachet is comparably higher and better for health than other common drinking water sources [25–28]. Instead, external contamination of the plastic sachet may be the mode of substantial pathogen transmission, as a recent study from Malawi suggests [29]. Water sachets are consumed by biting off an edge and sucking the water out of the sachet, thereby making direct and continuous contact between mouth and the sachet unavoidable. This mode of transmission was also hypothesized from an outbreak investigation in Accra, Ghana during a cholera outbreak in 2014. Among a population that relied heavily on sachet water as a source of drinking water, the investigators reported based on univariate analysis that consumption of this type of water was associated with a six-fold increase in cholera risk [30]. However, since no adjustment for potential confounding was done, the size of the reported effect should be interpreted with caution.

Funeral attendance and contact with symptomatic diarrhoea patients, variables with p-values between 0.1 and 0.05 in our study, are epidemiologically plausible risk factors for cholera infection and in line with other research [31,32]. Contact with a person showing symptoms of diarrhoea might have also functioned as a proxy of other unexplored risk factors in our study like shared contaminated water sources. On the other hand, other behavioural factors like place of food and fruit purchase or hygiene procedures prior to consumption that have been identified elsewhere [33] were not strongly associated with the outcome in our final model.

This was a field study carried out during an active outbreak, which led to certain limitations. First, not all included cases were laboratory-confirmed, and we do not know which ones. As per routine practice during cholera response, laboratory confirmation is stopped after an outbreak is detected and declared. This was also the case in Kinshasa, where only 117 sample from an overall 1,065 cases were sent for laboratory testing in the 2017–2018 outbreak [9]. Given the clear clinical picture of full-blown cholera, the fact that CTC admission criteria followed national and international standards [19,34], and that the triage staff were experienced health care workers should keep the proportion of false-positive inclusions among cases low. Second, taking stool samples from controls to rule out cholera infection was not feasible in our study, which might have led to an inclusion of some false inclusions among controls. Field enumerators followed standardized and detailed clinical questions to ascertain the eligibility of controls, which ruled out the inclusion of symptomatic persons among controls but not of asymptomatic infections. If present, both types of misclassifications would have led to an underestimation rather than an overestimation of the associations we detected in our study. Third, differentiating definitively between (internal) water contamination and (external) sachet contamination as mode of cholera transmission in this outbreak was not possible since this would have required systematic sampling and testing of water sachets along the production, retailing and consumption process. Forth, we did not asses vaccination status among participants from an oral cholera vaccination campaign done in 2016. However, given that most of our study area was not covered by that campaign and the fact that protective effects from cholera vaccines are not believed to last beyond two years, we believe that this did not affect our results substantially. Fifth, we only recruited 390 participants instead of our target sample size of 402, and some variables had missing data (Table 2). However, since the discrepancy between the actual and target sample size and the amount of missing data was not big, we do not believe that this affected our results substantially.

Noteworthy strengths of this study include prospective and systematic data collection using detailed SOPs and CRFs; the community-based design for recruitment of controls; the involvement of trained field epidemiologists as data collectors; and the application of advanced statistical methods to triangulate the validity of our findings.

Although cholera is an old disease with well-known classical risk factors, each setting is unique and produces its own context-specific transmission patterns and risk factors. Sachet water consumption has experienced a sharp rise during the past decade in SSA and is particularly common in cities rather than in rural areas [9,25,35,36]. With more than 80% of our study participants reporting the consumption of this type of water, Kinshasa is no exception to this phenomenon. Future behavioural cholera risk factors studies should ideally include mixed-methods approaches to contextualize findings and reveal other previously not considered modes of cholera transmission.

WHO’s 2030 roadmap, in which DRC features as a key country, and in particular DRC’s own cholera elimination agenda are very ambitious [7,16,37]. Early outbreak detection and quick response is the cornerstone of both strategies. For this, understanding local transmission patterns remains key, in particular in urban areas with particular risk behaviours for which existing evidence remains scarce. This is acknowledged in DRC’s current multisectoral strategy plan for the elimination of cholera, which explicitly recognises the need for continuous operational research to improve outbreak response interventions [17]. The use of oral cholera vaccination has gained momentum in recent years through the creation of a global stockpile and has, despite remaining challenges, become an important tool in the response to endemic and epidemic cholera [38,39]. However, as with other technical innovation, this must not come at the cost of traditional outbreak investigation and response measures that are informed through locally acquired contextual insights. For this, the role of field epidemiology will remain crucial. Only by adapting our variable selection and data collection tool to the local setting of Kinshasa and informed by local knowledge were able to identify sachet water consumption as an important risk factor in this outbreak.

Many parts of DRC had been free of cholera for many years. The nation-wide resurgence of cholera that started in 2015 in Eastern DRC, which ultimately led to the urban outbreak in Kinshasa described here, shows how easily gains can be lost. Our study highlights the need for and added value of tailored outbreak investigations as part of a comprehensive and successful response strategy.

In conclusion, the consumption of water from street-vended sachets needs to be considered as a potential route of cholera transmission in urban settings where the use of this drinking water type is frequent. Health messaging, outbreak control measures and epidemiological investigations should include the consumption of water sachets as a potential risk factors during future cholera outbreaks in urban low-resource contexts.

## Supporting information

S1 Table Variable definitions and measurement. Name, description and method of measurement of all variables.
(DOCX)

S2 Table Age distribution among study participants. Number and percentages of included study participants, pooled for cases and controls. N Numbers; % Percentage.
(DOCX)

S3 Table Sensitivity analysis using Complete Case Missing Value method. Parameter estimates and Type III analysis. SE Standard Error; OR Odds Ratio; CI Confidence Interval; DF Degrees of Freedom.
(DOCX)

S4 Table Sensitivity analysis using Subgroup Analysis method. Parameter estimates and Type III analysis. SE Standard Error; OR Odds Ratio; CI Confidence Interval; DF Degrees of Freedom.
(DOCX)

S5 Table Multicollinearity analysis. Inflation factor analysis. GVIF General Variance Inflation Factor; DF Degrees of Freedom.
(DOCX)
