# Supplementary material for: Sachet water consumption as a risk factor for cholera in urban settings: Findings from a case control study in Kinshasa, Democratic Republic of the Congo during the 2017–2018 outbreak
Source: PLoS Negl Trop Dis. 2021 Jul 8;15(7):e0009477. doi: 10.1371/journal.pntd.0009477 (PMC8266059; doi:10.1371/journal.pntd.0009477)
Supplement: S1 Table — Name, description and method of measurement of all variables. (DOCX) [file pntd.0009477.s001.docx]

S1 Table

Title: Variable definitions and measurement.

Description: Name, description and method of measurement of all variables.

| **Variable name in Tables 1 and 2** | **Detailed variable definition** | **Method of measurement** |
| --- | --- | --- |
| Sex | Sex of participant | response by participant |
| Religion | Religious denomination of participant | response by participant |
| Soil condition in area of residence | Soil condition in the area of residence | inspection by data collector |
| Level of education | Level of education of participant | response by participant |
| Occupation | Occupation of participant | response by participant |
| Household size | Number of persons living in same household as participant | response by participant |
| Presence of soap in the household | Presence of soap in the household of the participant | response by participant and inspection by data collector |
| Source of drinking water of the household | Source of drinking water of the participant's household | response by participant |
| Presence of toilet in the household | Presence of toilet and fecal waste in the participant's household | response by participant and inspection by data collector |
| Travelled outside Kinshasa since start of epidemic | Participant travelled outside Kinshasa since the start of the cholera epidemic | response by participant |
| Attended funeral since start of epidemic | Participant attended a funeral since the start of the cholera epidemic | response by participant |
| Health zone of area of residence | Heath zone to which the participant's area of residence belongs | local knowledge of data collector |
| Place of fruit/food purchase | Main place of fruit and food purchase of participant | response by participant |
| Contact with diarrheal patient since start of epidemic | Participant had physical contact with a diarrhoea patient since the start of the cholera epidemic | response by participant |
| Procedure before food consumption | Procedure usually done by participant before food consumption | response by participant |
| Roadside fruit consumption | Frequency of fruit consumption on the roadside by the participant | response by participant |
| Sachet water consumption | Frequency of sachet water consumption by the participant | response by participant |
| Drinking water storage | Typ of drinking water container used in the participant's household | response by participant |
| Roadside food consumption | Frequency of food consumption on the roadside by the participant | response by participant |
| Procedure before fruit consumption | Procedure usually done by participant before fruit consumption | response by participant |
