# Supplementary material for: Sachet water consumption as a risk factor for cholera in urban settings: Findings from a case control study in Kinshasa, Democratic Republic of the Congo during the 2017–2018 outbreak
Source: PLoS Negl Trop Dis. 2021 Jul 8;15(7):e0009477. doi: 10.1371/journal.pntd.0009477 (PMC8266059; doi:10.1371/journal.pntd.0009477)
Supplement: S2 Table — Number and percentages of included study participants, pooled for cases and controls. N Numbers; % Percentage. (DOCX) [file pntd.0009477.s002.docx]

S2 Table

Title: Age distribution among study participants

Description: Number and percentages of included study participants, pooled for cases and controls. N Numbers; % Percentage.

|  | **N** | **%** |
| --- | --- | --- |
| **Years of age** |  |  |
| <5 | 48 | 12.31 |
| 5 - ≤10 | 52 | 13.33 |
| 10 - ≤5 | 40 | 10.16 |
| 15 - ≤20 | 46 | 11.79 |
| 20 - ≤30 | 91 | 23.33 |
| 30 - ≤40 | 53 | 13.59 |
| >40 | 60 | 15.38 |
