# Supplementary material for: Sachet water consumption as a risk factor for cholera in urban settings: Findings from a case control study in Kinshasa, Democratic Republic of the Congo during the 2017–2018 outbreak
Source: PLoS Negl Trop Dis. 2021 Jul 8;15(7):e0009477. doi: 10.1371/journal.pntd.0009477 (PMC8266059; doi:10.1371/journal.pntd.0009477)
Supplement: S3 Table — Parameter estimates and Type III analysis. SE Standard Error; OR Odds Ratio; CI Confidence Interval; DF Degrees of Freedom. (DOCX) [file pntd.0009477.s003.docx]

S3 Table

Title: Sensitivity analysis using Complete Case Missing Value method

Description: Parameter estimates and Type III analysis. SE Standard Error; OR Odds Ratio; CI Confidence Interval; DF Degrees of Freedom.

Significance: indicated by an asterisk (*)

|  | **Level** | **Estimate** | **SE** | **OR (95% CI)** | **P-value** |
| --- | --- | --- | --- | --- | --- |
| Religion: catholic | 1 | 1.6 | 0.741 | 5.0 (1.2 - 21.6) | 0.029 |
| Religion: protestant | 2 | 1.3 | 0.749 | 3.7 (0.9 - 16.2) | 0.079 |
| Religion: revival | 3 | 1.2 | 0.638 | 3.3 (0.9 - 11.4) | 0.064 |
| Attended funeral recently: yes | 1 | 0.9 | 0.607 | 2.6 (0.8 - 8.5) | 0.120 |
| Procedure before fruit consumption: wipe with hands | 2 | 0.3 | 0.587 | 1.3 (0.4 - 4.1) | 0.657 |
| Procedure before fruit consumption: none | 3 | 0.4 | 0.411 | 1.5 (0.7 - 3.4) | 0.299 |
| Place of food/fruit purchase: roadside, street, restaurant | 2 | -0.2 | 0.371 | 0.8 (0.4 - 1.7) | 0.650 |
| Recent contact with diarrheal patient: yes | 1 | -0.5 | 0.247 | 0.6 (0.4 - 1.0) | 0.064 |
| Procedure before food consumption: none | 2 | -0.5 | 0.384 | 0.6 (0.3 - 1.3) | 0.201 |
| Level of education: primary | 1 | -0.7 | 0.359 | 0.5 (0.3 - 1.0) | 0.067 |
| Level of education: none | 3 | -0.7 | 0.452 | 0.5 (0.2 - 1.2) | 0.099 |
| Sachet water consumption: yes rarely | 2 | 0.7 | 0.453 | 2.1 (0.9 - 5.1) | 0.105 |
| Sachet water consumption: yes often | 3 | 1.3 | 0.457 | 3.8 (1.6 - 9.4) | 0.003* |
| Sachet water consumption: yes very often | 4 | 1.4 | 0.710 | 4.1 (1.0 - 16.5) | 0.047 |
| Source of drinking water: unprotected | 1 | -0.8 | 0.797 | 0.4 (0.1 - 2.1) | 0.308 |

|  | **DF** | **Wald Test** | **P-value** |
| --- | --- | --- | --- |
| Religion | 3 | 5.991 | 0.111 |
| Attended funeral recently | 1 | 2.960 | 0.085 |
| Procedure before fruit consumption | 2 | 2.080 | 0.352 |
| Place of food/fruit purchase | 1 | 0.281 | 0.598 |
| Recent contact with a diarrhoea patient | 1 | 3.480 | 0.061 |
| Procedure before food consumption | 1 | 2.301 | 0.128 |
| Level of education | 2 | 3.911 | 0.141 |
| Sachet water consumption | 3 | 9.311 | 0.025* |
| Source of drinking water | 1 | 1.136 | 0.286 |
