# Supplementary material for: Sachet water consumption as a risk factor for cholera in urban settings: Findings from a case control study in Kinshasa, Democratic Republic of the Congo during the 2017–2018 outbreak
Source: PLoS Negl Trop Dis. 2021 Jul 8;15(7):e0009477. doi: 10.1371/journal.pntd.0009477 (PMC8266059; doi:10.1371/journal.pntd.0009477)
Supplement: S4 Table — Parameter estimates and Type III analysis. SE Standard Error; OR Odds Ratio; CI Confidence Interval; DF Degrees of Freedom. (DOCX) [file pntd.0009477.s004.docx]

S4 Table

Title: Sensitivity analysis using Subgroup Analysis method

Description: Parameter estimates and Type III analysis. SE Standard Error; OR Odds Ratio; CI Confidence Interval; DF Degrees of Freedom.

Significance: indicated by an asterisk (*)

|  | **Level** | **Estimate** | **SE** | **OR (95% CI)** | **P-value** |
| --- | --- | --- | --- | --- | --- |
| Religion: catholic | 1 | 1.623 | 0.725 | 5.1 (1.2 – 21.0) | 0.025 |
| Religion: protestant | 2 | 1.327 | 0.742 | 3.8 (0.9 - 16.1) | 0.075 |
| Religion: revival | 3 | 1.172 | 0.627 | 3.2 (0.9 - 11.0) | 0.062 |
| Attended funeral recently: yes | 1 | 0.936 | 0.606 | 2.5 (0.8 - 8.4) | 0.123 |
| Procedure before fruit consumption: wipe with hands | 2 | 0.034 | 0.602 | 1.0 (0.3 - 3.4) | 0.955 |
| Procedure before fruit consumption: none | 3 | 0.270 | 0.372 | 1.3 (0.6 - 2.7) | 0.470 |
| Place of food/fruit purchase: roadside, street, restaurant | 1 | -0.194 | 0.359 | 0.8 (0.4 - 1.7) | 0.591 |
| Recent contact with diarrheal patient: yes | 1 | -0.454 | 0.247 | 0.6 (0.4 - 1.0) | 0.066 |
| Procedure before food consumption: none | 2 | -0.479 | 0.374 | 0.6 (0.3 - 1.3) | 0.201 |
| Level of education: primary | 1 | -0.662 | 0.359 | 0.5 (0.3 - 1.0) | 0.065 |
| Level of education: none | 3 | -0.753 | 0.451 | 0.5 (0.2 - 1.1) | 0.095 |
| Sachet water consumption: yes rarely | 2 | 0.729 | 0.447 | 2.1 (0.9 – 5.0) | 0.103 |
| Sachet water consumption: yes often | 3 | 1.329 | 0.460 | 3.8 (1.5 - 9.3) | 0.004* |
| Sachet water consumption: yes very often | 4 | 1.411 | 0.694 | 4.1 (1.1 – 16.0) | 0.042 |
| Source of drinking water: unprotected | 1 | -0.813 | 0.787 | 0.4 (0.1 - 2.1) | 0.301 |

|  | **DF** | **Wald Test** | **P-value** |
| --- | --- | --- | --- |
| Religion | 3 | 6.051 | 0.108 |
| Attended funeral recently | 1 | 2.950 | 0.085 |
| Procedure before fruit consumption | 2 | 2.490 | 0.115 |
| Place of food/fruit purchase | 1 | 0.382 | 0.531 |
| Recent contact with a diarrhoea patient | 1 | 3.522 | 0.060 |
| Procedure before food consumption | 1 | 2.491 | 0.521 |
| Level of education | 2 | 4.020 | 0.134 |
| Sachet water consumption | 3 | 9.411 | 0.028* |
| Source of drinking water | 1 | 1.134 | 0.287 |
