# Supplementary material for: Sachet water consumption as a risk factor for cholera in urban settings: Findings from a case control study in Kinshasa, Democratic Republic of the Congo during the 2017–2018 outbreak
Source: PLoS Negl Trop Dis. 2021 Jul 8;15(7):e0009477. doi: 10.1371/journal.pntd.0009477 (PMC8266059; doi:10.1371/journal.pntd.0009477)
Supplement: S5 Table — Inflation factor analysis. GVIF General Variance Inflation Factor; DF Degrees of Freedom. (DOCX) [file pntd.0009477.s005.docx]

S5 Table

Title: Multicollinearity analysis

Description: Inflation factor analysis. GVIF General Variance Inflation Factor; DF Degrees of Freedom.

|  | **GVIF** | **DF** | **GVIF^(1/2*DF))** |
| --- | --- | --- | --- |
| Religion | 3.338 | 3 | 1.222 |
| Level of education | 4.642 | 2 | 1.468 |
| Attended funeral recently | 4.925 | 1 | 2.219 |
| Sachet water consumption | 4.505 | 3 | 1.285 |
| Procedure before fruit consumption | 5.034 | 2 | 1.498 |
| Place of food/fruit purchase | 2.384 | 1 | 1.544 |
| Recent contact with a diarrhoea patient | 1.791 | 1 | 1.338 |
| Procedure before food consumption | 6.482 | 1 | 2.546 |
| Source of drinking water | 1.000 | 1 | 1.000 |
